# Thioredoxin 1 supports colorectal cancer cell survival and promotes migration and invasion under glucose deprivation through interaction with G6PD

**DOI:** 10.7150/ijbs.71809

**Published:** 2022-08-29

**Authors:** Fengying Lu, Daoquan Fang, Shuhan Li, Zuyue Zhong, Xiujiao Jiang, Qinqin Qi, Yining Liu, Wenqi Zhang, Xiaohui Xu, Yangyang Liu, Weijian Zhu, Lei Jiang

**Affiliations:** 1 Central Laboratory, the First Affiliated Hospital of Wenzhou Medical University, Wenzhou 325000, China.; 2 Changzhou maternal and Child Health Care Hospital, Changzhou Medical Center, Nanjing Medical University, Changzhou, 213000, China.

**Keywords:** Thioredoxin 1, G6PD, Glucose deprivation, Colorectal cancer

## Abstract

Overcoming energy stress is a critical step for cells in solid tumors. Under this stress microenvironment, cancer cells significantly alter their energy metabolism to maintain cell survival and even metastasis. Our previous studies have shown that thioredoxin-1 (Trx-1) expression is increased in colorectal cancer (CRC) and promotes cell proliferation. However, the exact role and mechanism of how Trx-1 is involved in energy stress are still unknown. Here, we observed that glucose deprivation of CRC cells led to cell death and promoted the migration and invasion, accompanied by upregulation of Trx-1. Increased Trx-1 supported CRC cell survival under glucose deprivation. Whereas knockdown of Trx-1 sensitized CRC cells to glucose deprivation-induced cell death and reversed glucose deprivation-induced migration, invasion, and epithelial-mesenchymal transition (EMT). Furthermore, we identified glucose-6-phosphate dehydrogenase (G6PD) interacting with Trx-1 by HuPortTM human protein chip, co-IP and co-localization. Trx-1 promoted G6PD protein expression and activity under glucose deprivation, thereby increasing nicotinamide adenine dinucleotide phosphate (NADPH) generation. Moreover, G6PD knockdown sensitized CRC cells to glucose deprivation-induced cell death and suppressed glucose deprivation-induced migration, invasion, and EMT. Inhibition of Trx-1 and G6PD, together with inhibition of glycolysis using 2-deoxy-D-glucose (2DG), resulted in significant anti-tumor effects in CRC xenografts *in vivo*. These findings demonstrate a novel mechanism and may represent a new effective therapeutic regimen for CRC.

## Introduction

Colorectal cancer (CRC) is one of the most common causes of cancer-related death worldwide 1, 2. The tumor microenvironment plays an important role in tumor development and progression, and thus may be a potential target for chemoprevention and therapy 3. A previous study reported that the cellular metabolism within a solid tumor is significantly different from that of the corresponding normal tissue 4. One of the most known causes of altered tumor metabolism is the unique microenvironmental stresses that exist in tumors. Because of the poor vascularization of the tumor, its cells are exposed to a fluctuating microenvironment with limited supply of oxygen and glucose 5. For example, glucose concentration in human colon and gastric cancer tissues is significantly lower than in surrounding non-cancerous tissues 6, 7. Consequently, tumor cells can overcome these unfavorable growth conditions through mechanisms of metabolic adaptation.

Thioredoxin-1 (Trx-1), a potent protein disulfide reductase, plays a critical role in regulating cellular redox status 8. Trx-1 is induced by various oxidative stresses and regulates numerous cellular processes, including cell proliferation, apoptosis, antioxidant response, and gene expression 9. There is increasing evidence that Trx-1 plays an important role in tumor progression and metastasis. In our previous studies, we found that overexpression of Trx-1 promotes invasion and metastasis of CRC cells, and conversely, suppression of Trx-1 expression inhibits invasion and metastasis of CRC cells 10, 11. However, the function of Trx-1 in the survival and metastasis of CRC cells under metabolic stress and its mechanism of action remain to be elucidated.

Aerobic glycolysis (or the Warburg effect) has been recognized as a hallmark of cancer. This metabolic signature allows cancer cells to use glucose for biosynthesis, which supports their high proliferation requirements 12. Glucose flux into the pentose phosphate pathway (PPP) is the main source for the generation of NADPH 13, the major intracellular reducing agent required for reductive biosynthesis 14, 15. Moreover, NADPH is essential for ROS scavenging and thus crucial for antioxidant defense in cancer cells 16. Therefore, maintaining adequate NADPH levels is key to the survival of cancer cells under oxidative stress.

Glucose-6-phosphate dehydrogenase (G6PD), the first key enzyme of PPP, is a central enzyme that generates cellular ribose and the reducing equivalent NADPH 17. These two molecules are necessary for many processes related to biosynthesis and reductive biosynthesis, such as lipogenesis, antioxidative stress and cell growth. G6PD is overexpressed in various tumors 18-20, and is considered the main control point for NADPH production in cancer cells 17, 21. Notably, disruption of NADPH production can increase cell sensitivity to ROS and induce apoptosis 21, 22.

In the present study, we discovered a novel protective function of Trx-1 in CRC cells under glucose deprivation. We demonstrated that Trx-1 regulates the activity of G6PD by interacting with G6PD, which increases NADPH generation *via* PPP and maintains the survival of CRC cells. In addition, Trx-1 and G6PD are required for CRC cell migration, invasion and epithelial-to-mesenchymal transition (EMT) under the glucose-starved condition. Inhibition of Trx-1 and G6PD in combination with inhibition of glycolysis using 2-deoxy-D-glucose (2DG) resulted in a substantial anti-tumor effect in CRC xenografts *in vivo*. Our current study provides a novel mechanism for the involvement of Trx-1 in surviving glucose starvation *via* G6PD-mediated NADPH homeostasis in CRC cells.

## Materials and methods

### Cell culture and treatments

Human CRC cell lines SW480 and SW620 were purchased from the Type Culture Collection of the Chinese Academy of Sciences (Shanghai, China). SW620 cells were maintained in RPMI 1640 supplemented with 10% fetal bovine serum (FBS) (Thermo Fisher Scientific, Waltham, MA, USA). SW480 cells were maintained in Dulbecco's Modified Eagle's Medium (DMEM) (Thermo Fisher Scientific) supplemented with 10% FBS. Cells were cultured in a humidified 37°C incubator with 5% CO_2_. For glucose deprivation experiments, cells were washed with PBS and cultured in glucose-free medium supplemented with 10% dialysed FBS (Thermo Fisher Scientific) 23.

### Cell transfection and lentivirus production

Human G6PD siRNA (HSS103893, 5'- AAACCCACUCUCUUCAUCAGCUC GU -3') was purchased from Invitrogen (Carlsbad, CA). Full-length G6PD DNA fragments were amplified by polymerase chain reaction (PCR) from G6PD/pRK5 plasmid (#41521, Addgene, Watertown, MA, USA), and cloned into pCMV-3Tag-1 vector (Sigma). The Myc-tagged Trx-1 plasmid was obtained from Addgene (#216714). Cells were transiently transfected with siRNA or plasmid DNA using Lipofectamine 3000 (Thermo Fisher Scientific) according to the manufacturer's protocol. Lentiviral vectors containing the human Trx-1 gene or Trx-1 shRNA were cloned as previously described 10, 11. Lentiviral vectors carrying green fluorescent protein (GFP) gene or shRNA targeting firefly luciferase were used as controls (lenti-GFP or shLuc) 10. Lentivirus production and transduction were performed as previously described 10.

### Migration and invasion assay

Cell migration ability was analyzed using Transwell inserts with 8 µm pores (Corning Costar Corp., Cambridge, MA, USA). Medium containing 20% FBS (600 µL) in the lower chamber of each well was used as chemoattractant. The upper wells were filled with 2.5 × 10^4^ cells in 150 μl of medium without serum and glucose. After an incubation period of 48 h, the insert was stained with 0.1% crystal violet, and the non-migrated cells were wiped from the upper surface of the membrane with a cotton swab. For the invasion assay, the polycarbonate membranes of the Transwell inserts were pre-coated with 10 µL matrigel (BD Biosciences, San Jose, CA, USA). When performing the invasion assay, the upper compartments were seeded with 1 × 10^5^ cells. Each assay was carried out in triplicate. The average number of migrating or invading cells was counted in five random microscope fields.

### Cell death, colony formation, and apoptosis assays

Cell death was detected by trypan blue staining. Colony formation assay was performed by subjecting cells to glucose deprivation and then growing them for 14 days under normal condition 24. Cell apoptosis was determined using an Annexin V-FITC/propidium iodide (PI) apoptosis kit (Multisciences Biotech Co., Ltd., China) according to the manufacturer's instructions. The percentage of cell apoptosis was analyzed by flow cytometry (CytoFlex LX; Beckman Coulter).

### Western blot analysis

Cells were lysed with lysis buffer (P89901; Thermo Scientific) containing protease inhibitors (P8340; Sigma-Aldrich). Western blot analysis was performed as previously described 10. The following primary antibodies were used: anti-Trx-1 (1: 1000), anti-G6PD (1:2000), and GAPDH (1:5000) from Abcam (Cambridge, UK); anti-E-cadherin (1:1000; Cell Signaling Technology, Beverly, MA), and anti-vimentin (1:5000; Santa Cruz, CA, USA). Protein band intensity was quantified using Image Lab Software (Bio-Rad Laboratories Inc., Berkeley, CA, USA).

### Co-immunoprecipitation (Co-IP)

Antibodies with the Myc tag (Cat. #ab206486, Abcam, Cambridge, UK) and Flag tag (Cat. # ab66008-2; Proteintech, Rosemont, IL, USA) were used in coimmunoprecipitation assays. The total protein extract (500 μg) was incubated with the primary antibody (1 μg) or IgG at 4°C for 2 h. The mixture was then incubated with SureBeads Protein G Magnetic Beads (Bio-Rad Laboratories Inc.) at 4°C for 1 h. The beads were then collected and eluted with 2 × sample loading buffer. The eluted samples were boiled for 5 min and analyzed by Western blotting.

### Immunofluorescence assays

Cells plated on coverslips were fixed with 4% paraformaldehyde and then permeabilized with 0.1% Triton X-100. After incubation in 5% BSA for 30 min, the slides were incubated overnight at 4°C with anti-Trx-1 (1:100) or anti-G6PD (1:200) antibodies. Subsequently, the slides were incubated with either Alexa Fluor Plus 594 goat anti-rabbit IgG antibody or Alexa Fluor Plus 488 goat anti-mouse IgG antibody (Invitrogen) for 1 h. The nuclei were then stained with DAPI solution. Images were captured using a confocal microscope (Leica SP8, Germany).

### ROS detection assay

Intracellular ROS level was detected by a ROS assay kit using DCFH-DA as a molecular probe (Nanjing Jiancheng Bioengineering Institute, Nanjing, China). After glucose starvation treatment, cells were harvested and incubated with 10 μM DCFH-DA for 20 min at 37°C. Cell fluorescence was analyzed using CytoFlex LX flow cytometer (Beckman Coulter). In addition, the level of ROS in GFP expressing cells was detected using the Cellular Reactive Oxygen Species Detection Assay Kit (Deep Red Fluorescence, Abcam) according to the manufacturer's instructions.

### G6PD enzyme activity

G6PD enzyme activity was measured using the Glucose 6 Phosphate Dehydrogenase Activity Assay Kit (Abcam, ab176722) according to the manufacturer's instructions. In brief, cells (5 × 10^5^) were harvested and washed with cold PBS. The cells were then lysed with Mammalian Cell Lysis Buffer (Abcam, ab179835) for 10 min and centrifuged to remove insoluble material. The supernatant (50 μl) was transferred to a 96-well plate and 50 μl of G6PD Assay Mixture was added to each well. Fluorescence was measured at Ex/Em = 540/590 nm using SpectraMax Microplate Reader (Molecular Devices, San Jose, CA, USA), and G6PDH activity was calculated according to the manufacturer's instructions.

### Determination of NADPH levels

The colorimetric NADP/NADPH Quantitation Kit (Biovision Inc., Milpitas, CA, USA) was used to detect the NADPH levels. The absorbance at 450 nm was read and normalized to the number of cells.

### Animal model experiments

Male BALB/c nude mice (3-4 weeks old, 16-20 g) were purchased from Beijing Vital River Laboratory Animal Technology Co., Ltd. (Beijing, China). The mice were given one week to acclimate to the new laboratory conditions before the experimentation. The CRC xenograft model was prepared by subcutaneous injection of 5 × 10^6^ SW480 cells into the right flank of mice. After tumors were measured on day 7, mice were randomly divided into six treatment groups (n = 5): Control (vehicle), 2DG (400 mg/kg), dehydroepiandrosterone (DHEA; 60 mg/kg), PX-12 (12 mg/kg), 2DG + DHEA, and 2DG + DHEA + PX-12. Mice were treated with intraperitoneal injections of the corresponding drug three times per week for five weeks. During the experiment, tumor length and width were measured every three days using a caliper, and volume was calculated using the formula (0.5 × length × width^2^). All animal experiments were approved by Laboratory Animal Ethics Committee of the First Affiliated Hospital of Wenzhou Medical University.

### Bioinformatics analysis

The differences of gene expression between tumor and normal samples were visualized using the Gene Expression Profiling Interactive Analysis (GEPIA2) database (http://gepia2.cancer-pku.cn/) 25, which contains TCGA data and GTEx data 26, 27. Count data from the TCGA-COAD and TCGA-READ datasets were downloaded from the UCSC Xena project (https://xena.ucsc.edu) 28 and used to perform correlation analysis using the Spearman method.

### Statistical analysis

Data are presented as mean ± standard error of mean (SEM). Enumeration data were compared using the chi-square test, and continuous data were compared with an independent *t*-test (SPSS, Chicago, IL, USA). A *p-*value of less than 0.05 was considered statistically significant.

## Results

### Glucose deprivation induces cell death and enhances cell migration and invasion in CRC cells

**Figure 1:**
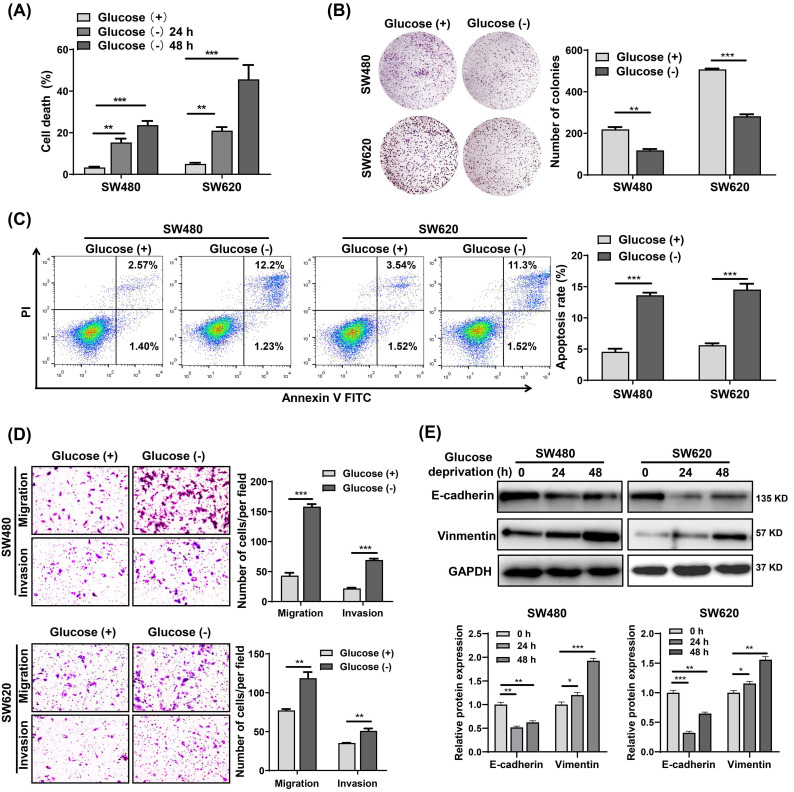
Glucose deprivation induces cell death and enhances cell migration and invasion in colorectal cancer cells. (A) Cell death was assessed by trypan blue assays in SW480 and SW620 cells grown for 24 h and 48 h without glucose. (B) Colony formation assays were performed in SW480 and SW620 cells after 24 h of glucose deprivation. (C) The percentage of apoptosis was determined by flow cytometry using Annexin V/propidium iodide (PI) staining in SW480 and SW620 cells after 24 h of glucose-deprivation. (D) Cell migration and invasion ability was measured by Transwell assays. SW480 and SW620 cells were treated with glucose-free medium for 24 h. (E) E-cadherin and vimentin expressions were determined by Western blot analysis in SW480 and SW620 cells treated with glucose-deprivation medium for 24 h and 48 h, respectively. GAPDH was used as a loading control. Data are presented as mean ± SEM (n = 3). *p < 0 05, **p < 0 01, and ***p < 0 001.

To investigate the effects of metabolic stress on CRC cells survival, we cultured SW480 and SW620 cells in glucose starvation conditions. The results showed that glucose deprivation significantly induced SW480 and SW620 cell death in a time-dependent manner (**Fig. 1A**). However, glucose deprivation did not significantly affect the survival of FHC cells (**Fig. S1A**), suggesting that normal cells are significantly less susceptible to glucose deprivation-induced cytotoxicity compared with cancer cells. Also, the colony formation assay revealed that glucose metabolic stress led a significant decrease in the proliferative capability of SW480 and SW620 cells (**Fig. 1B**). Cell apoptosis analysis showed that the glucose deprivation treatment group had an apoptosis rate approximately three times higher than that of the control group (**Fig. 1C**). Moreover, Transwell assays showed that glucose deprivation promoted the migration and invasion ability of SW480 and SW620 cells (**Fig. 1D**). **Fig.1E** illustrates the decreased expression of E-cadherin and increased expression of vimentin observed in glucose deprived cells. These results suggest that glucose deprivation induces cell death and enhances cell migration and invasion in CRC cells.

### Glucose deprivation induces cell death via ROS accumulation in CRC cells

**Figure 2:**
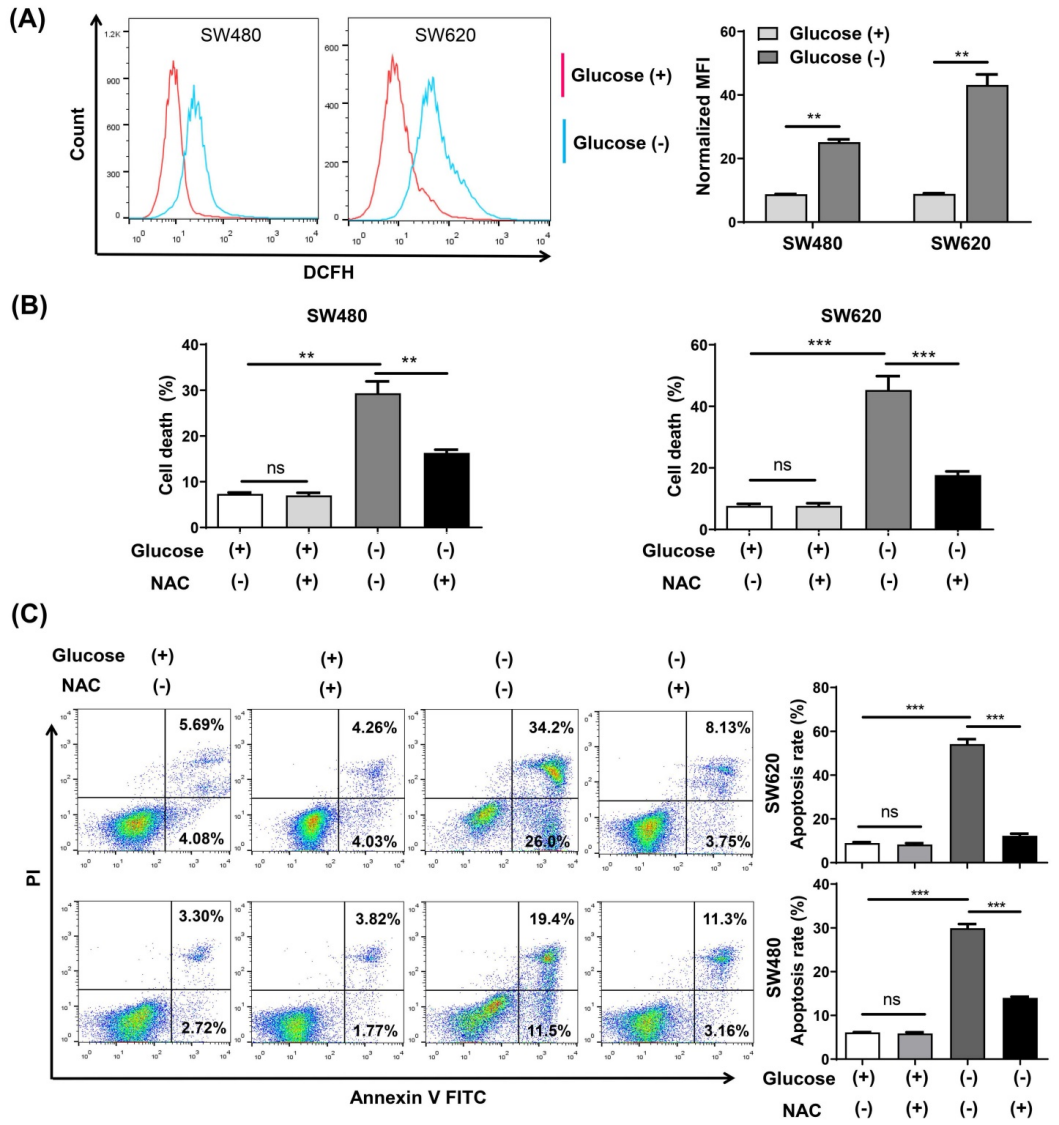
The ROS inhibitor N-acetyl-l-cysteine (NAC) reverses glucose deprivation-induced cell death of colorectal cancer cells. (A) Intracellular ROS levels were detected by flow cytometry using the fluorescent probe DCFH-DA in SW480 and SW620 cells incubated in glucose-free medium for 24 h. (B) Cell death was assessed by trypan blue assays in SW480 and SW620 cells cultured for 48 h in glucose-free medium in the presence or absence of NAC (5 mM). (C) The percentage of apoptosis was assessed by flow cytometry with Annexin V/propidium iodide (PI) staining in SW480 and SW620 cells cultured for 48 h in glucose-free medium in the presence or absence of NAC (5 mM). Data are expressed as mean ± SEM; n = 3. ns: not significant, ∗∗*p* < 0 01, and ∗∗∗*p* < 0 001.

Intracellular ROS changes were measured using flow cytometry analysis with the ROS fluorescence probe DCFH-DA. The results confirmed that glucose deprivation significantly increased ROS levels in SW480 and SW620 cells (**Fig. 2A**). We then subjected both cells lines to glucose deprivation for 48 h in the presence of the reactive oxygen scavenging agent N-acetyl-L-cysteine (NAC), and found that NAC increased cell survival and reversed the apoptosis caused by glucose deprivation (**Fig. 2B and 2C**).

### Increased Trx-1 supports CRC cells survival under glucose deprivation

**Figure 3:**
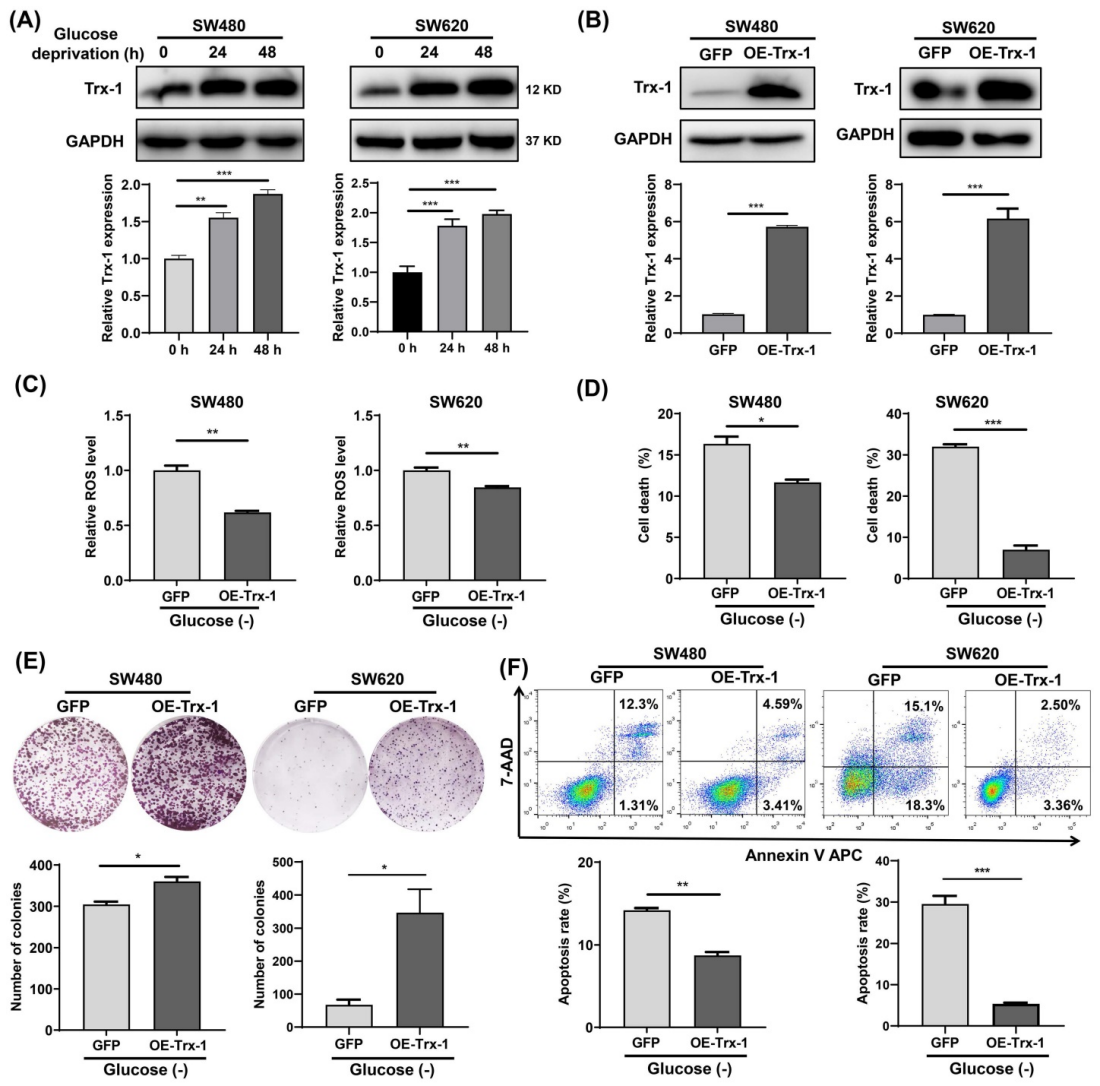
Increased Trx-1 supports survival of colorectal cancer cells under glucose deprivation. (A) Protein expression of Trx-1 was detected by Western blot analysis in SW480 and SW620 cells incubated for 24 h and 48 h in glucose-free medium. (B) Validation of Trx-1 overexpression by Western blot analysis. (C) The ROS levels in SW480 and SW620 cells stably expressing GFP or Trx-1 after incubation in glucose-deprived medium for 24 h. (D) Cell death assessed by trypan blue assays in SW480 and SW620 cells stably expressing GFP or Trx-1, after incubation in glucose-deprived medium for 24 h. (E) Colony formation assay of SW480 and SW620 cells stably expressing GFP or Trx-1 incubated in glucose-deprived medium for 24 h. (F) Cell apoptosis assessed by flow cytometry with Annexin V staining in SW480 and SW620 cells stably expressing GFP or Trx-1 cultured for 24 h in glucose-free medium. Data are shown as mean ± SEM; n = 3. ∗*p* < 0 05, ∗∗*p* < 0 01, and ∗∗∗*p* < 0 001.

Since Trx-1 is one of the most important members of the TRX antioxidant system, we examined its protein expression in glucose-deprived CRC cells by Western blotting. The results showed that glucose-deprivation treatment increased Trx-1 protein level in SW480 and SW620 cells (**Fig. 3A**). Next, we constructed stable cell lines overexpressing Trx-1 (SW480-OE-Trx-1 and SW620-OE-Trx-1) by means of lentivirus-mediated gene transfer. Increased level of Trx-1 protein in SW480 and SW620 cells transduced with lenti-Trx-1 was verified by Western blotting (**Fig. 3B**). ROS detection assays demonstrated that after glucose deprivation, intracellular ROS levels in cells overexpressing Trx-1 were significantly lower than in the control cells (**Fig. 3C**). Moreover, overexpression of Trx-1 in SW480 and SW620 cells promoted resistance to glucose deprivation-induced cell death, as evidenced by trypan blue exclusion assay and colony formation assay (**Fig. 3D and 3E**). Finally**,** overexpression of Trx-1 also promoted resistance to the cell apoptosis induced by glucose-deprivation (**Fig. 3F**).

### Knockdown of Trx-1 sensitizes CRC cells to glucose deprivation induced cell death

**Figure 4:**
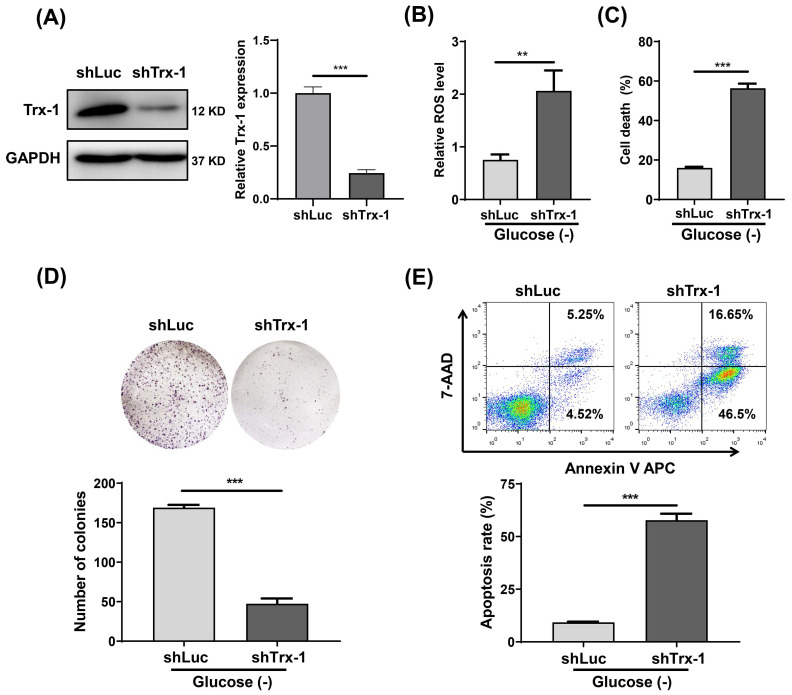
Knockdown of Trx-1 sensitizes colorectal cancer cells to glucose deprivation-induced cell death. (A) Trx-1 protein expression detected by Western blotting in SW480 cells stably expressing shLuc or shTrx-1. (B)The ROS level in SW480 cells stably expressing shLuc or shTrx-1, after 24 h incubation in glucose-deprived medium. (C) Cell death assessed by the trypan blue assays in SW480 cells stably expressing shLuc or shTrx-1 incubated in glucose-deprived medium for 24 h. (D) Colony formation assay of SW480 cells stably expressing shLuc or shTrx-1 after incubation for 24 h in glucose-deprived medium. (E) Cell apoptosis assessed by flow cytometry with Annexin V staining in SW480 cells stably expressing shLuc or shTrx-1 incubated in glucose-deprived medium for 24 h. Data are shown as mean ± SEM; n = 3. ∗∗*p* < 0 01, and ∗∗∗*p* < 0 001.

We constructed a SW480 cell line with stable Trx-1 knockdown using a lentiviral vector carrying shRNA targeting Trx-1 (shTrx-1). Decreased Trx-1 protein level in SW480 cells transfected with lenti-shTrx-1 was confirmed by Western blotting (**Fig. 4A**). ROS detection assays likewise demonstrated that after glucose deprivation, intracellular ROS levels were significantly higher in SW480 cells with Trx-1 knockdown than in control cells (**Fig. 4B**). In addition, knockdown of Trx-1 significantly increased glucose deprivation-induced cell death (**Fig.4C**). Finally, cell apoptosis and plate colony formation assays showed that Trx-1 knockdown sensitized SW480 cells to glucose deprivation (**Fig.4D and 4E**). Moreover, we knocked down Trx-1 expression in normal FHC cells and another CRC cell line SW620, and found that knockdown of Trx-1 also increased the sensitivity to glucose deprivation-induced cytotoxicity (**Fig.S1B-D and S2**).

### Knockdown of Trx-1 suppresses glucose deprivation-induced migration, invasion, and EMT in CRC cells

**Figure 5:**
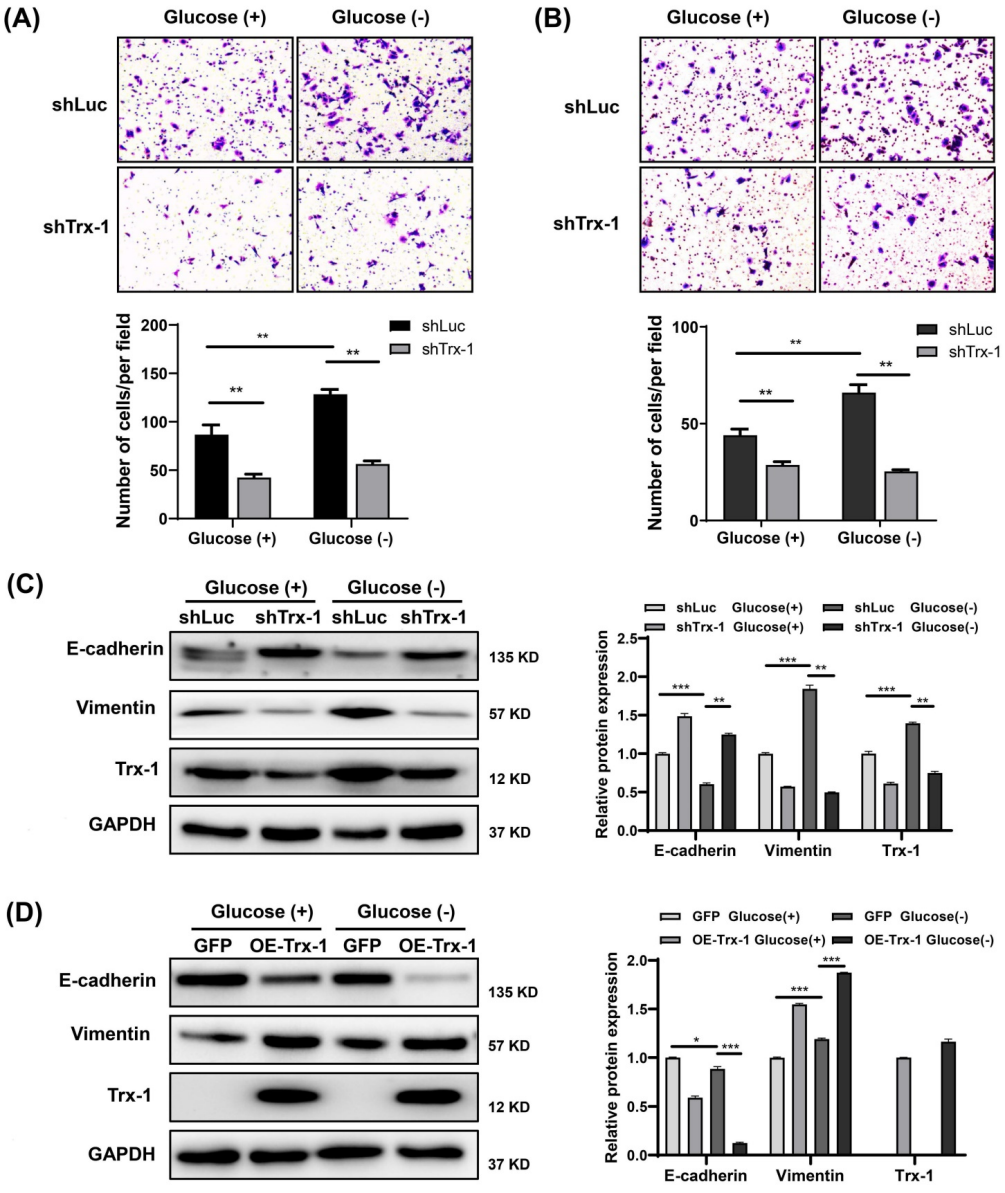
Knockdown of Trx-1 suppresses glucose deprivation-induced migration, invasion, and epithelial-mesenchymal transition in colorectal cancer cells. (A-B) Cell migration (A) and invasion (B) ability detected by Transwell assays in SW480 cells stably expressing shLuc or shTrx-1 after 24 h incubation in glucose-deprived medium. Representative images from triplicate experiments are shown. Magnification (200×). (C) Protein expressions of E-cadherin, vimentin, and Trx-1 as determined by Western blotting in SW480 cells stably expressing shLuc or shTrx-1, after incubation in complete medium or glucose-deprived medium for 24 h. GAPDH was used as a loading control. (D) Protein expressions of E-cadherin, vimentin, and Trx-1 determined by Western blotting in SW480 cells stably expressing GFP or Trx-1, after incubation in complete medium or glucose-deprived medium for 24 h. Data are shown as mean ± SEM; n = 3. ∗*p* < 0 05, ∗∗*p* < 0 01, and ∗∗∗*p* < 0 001.

Our previous study reported that Trx-1 promotes EMT, migration and invasion in CRC cells 10. As expected, Trx-1 knockdown reversed glucose deprivation-induced migration, invasion, and EMT in SW480 cells (**Fig. 5A-C**). Moreover, overexpression of Trx-1 promoted cell EMT with or without glucose deprivation (**Fig. 5D**).

### Trx-1 interacts with G6PD and increases its activity

**Figure 6:**
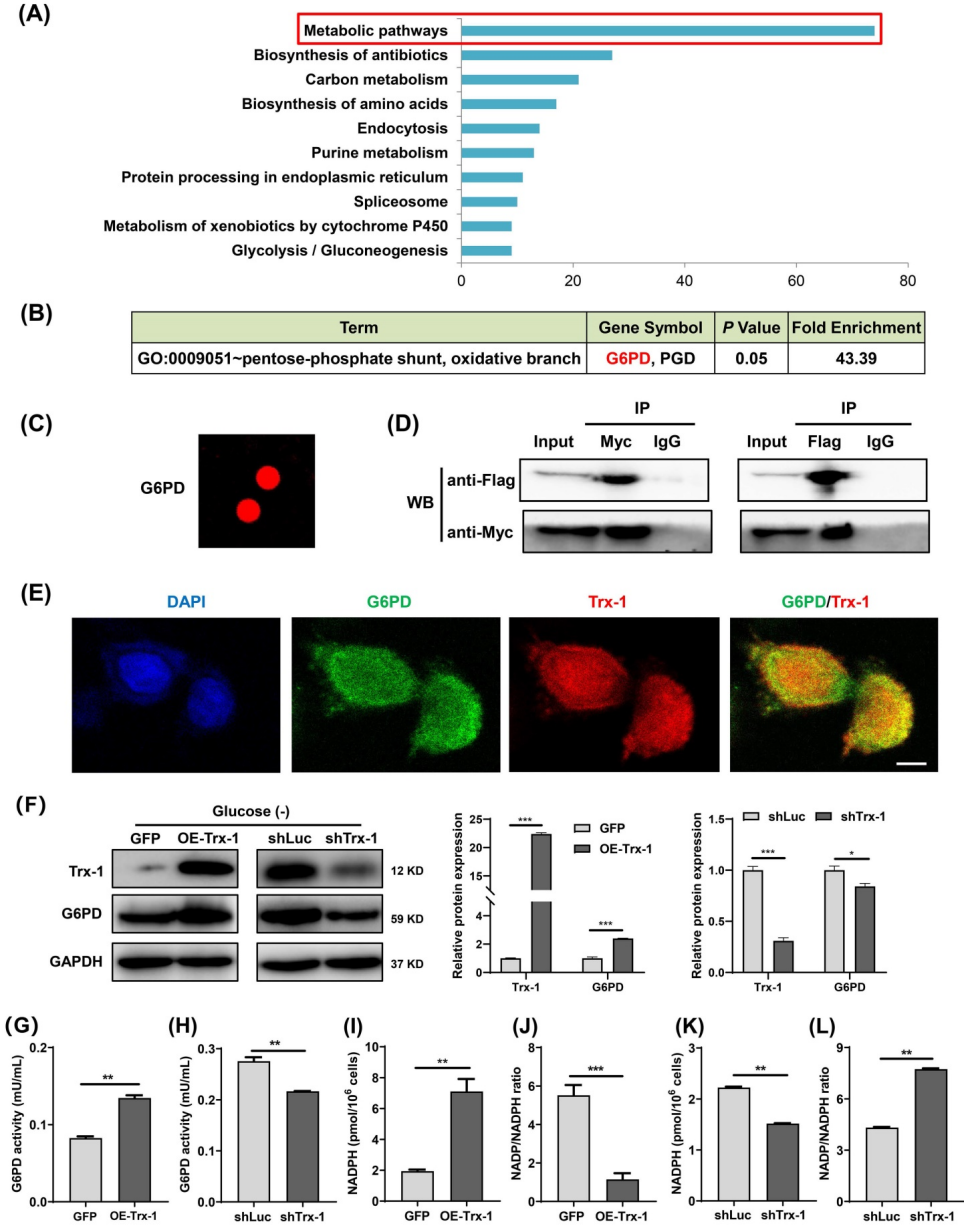
Trx-1 interacts with G6PD and increases its activity. (A) Protein interactors of “Trx-1” were identified using the HuPort^TM^ human protein chip, and the enrichment of related pathways was determined with reference to GO and KEGG pathways. (B) The HuPort^TM^ human protein chip identified G6PD as an interactor of Trx-1. (C) Depiction of the binding of Trx-1 with G6PD as identified by the HuPort^TM^ human protein chip. (D) The interaction between Trx-1 and G6PD was confirmed by co-IP assays. HEK293T cells transfected with Flag-G6PD and Myc-Trx-1 were immunoprecipitated with anti-Flag-tag or anti-Myc-tag antibodies, IgG was used as negative control. (E) SW480 cells were incubated under glucose starvation conditions for 24 h, and then cellular localization of Trx-1 and G6PD was determined by immunofluorescence staining. DAPI staining was performed to identify the nucleus. This fluorescence image was captured using a confocal microscope. The scale bar represents 25 μm. (F) Protein expressions of G6PD and Trx-1 determined by Western blotting in SW480 cells stably expressing GFP, Trx-1, shLuc or shTrx-1, after 24 h incubation in glucose-deprived medium. (G-H) G6PD activity in SW480 cells with Trx-1 overexpression or knockdown. (I-L) NADPH levels and NADP/NADPH ratio in SW480 cells with Trx-1 overexpression or knockdown. Data are shown as mean ± SEM; n = 3. ∗∗*p* < 0 01, and ∗∗∗*p* < 0 001.

To explore the mechanism underlying the support of CRC cells survival under glucose deprivation by Trx-1, its interacting proteins were identified using the HuPort^TM^ human protein chip. The results revealed 445 proteins binding to the Trx-1 protein. GO and KEGG pathway enrichment analysis indicated that Trx-1 interactors are significantly related to the “Metabolic Pathway” (**Fig. 6A**), with the highest enrichment being observed for G6PD, which is the key rate-limiting enzyme of the PPP (**Fig. 6B and 6C**). Subsequently, co-IP experiments were performed to confirm the interactions between Trx-1 and G6PD. As illustrated in **Fig.6D**, the results provide evidence of interaction between these two proteins. In addition, glucose deprivation increased the interaction between Trx-1 and G6PD in SW480 cells (**Fig S3A**). Immunofluorescence assays also indicated Trx-1 and G6PD colocalized in SW480 cells (**Fig. 6E**).

Moreover, we found that Trx-1 overexpression increased G6PD protein expression, whereas Trx-1 knockdown decreased its protein expression under glucose deprivation (**Fig. 6F**), but not in normal culture (**Fig.S3B**). Next, we investigated whether Trx-1 afffects the activity of G6PD. We observed that G6PD activity was increased by Trx-1 overexpression and significantly suppressed by its silencing (**Fig. 6G and 6H**). As mentioned above, G6PD is the rate-limiting enzyme of the PPP, which reduces equivalent reduced NADPH for reductive biosynthesis of lipids. Since Trx-1 can regulate the activity of this key control point of the PPP, we measured NADPH levels under conditions of Trx-1 overexpression or knockdown. In the setting of glucose deprivation, overexpression of Trx-1 increased the production of NADPH (**Fig. 6I**) and decreased the NADP/NADPH ratio (**Fig. 6J**); conversely, suppression of Trx-1 dramatically decreased the production of NADPH and increased the NADP/NADPH ratio (**Fig. 6K and 6L**).

### G6PD knockdown sensitizes CRC cells to glucose deprivation-induced cell death and suppresses glucose deprivation-induced cell migration, invasion, and EMT

**Figure 7:**
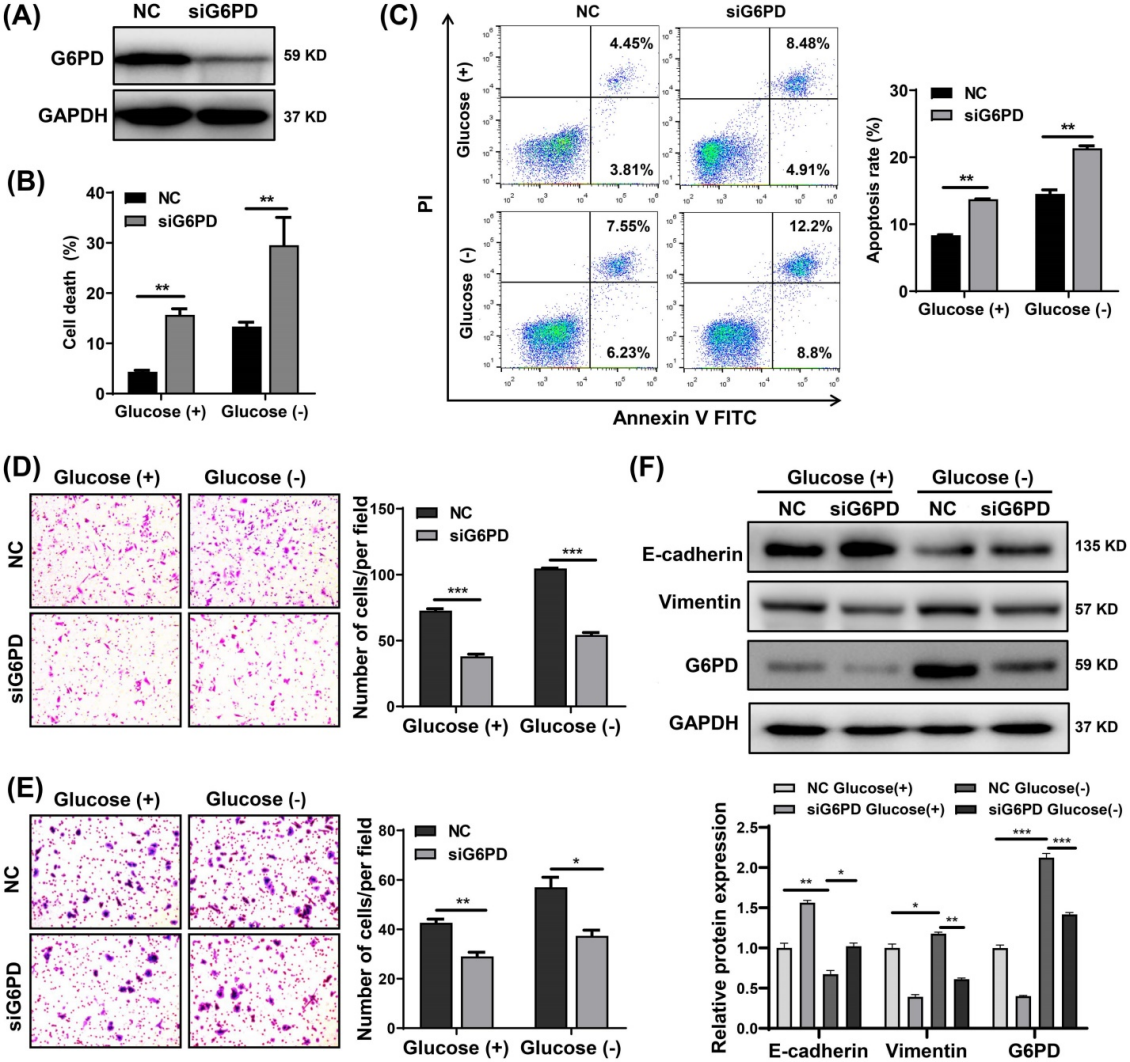
Knockdown of G6PD sensitizes colorectal cancer cells to glucose deprivation-induced cell death and suppresses glucose deprivation-induced migration, invasion, and epithelial-mesenchymal transition. (A) G6PD protein expressions determined by Western blotting in SW480 cells transfected with negative control (NC) or siG6PD (B) Cell death assessed by trypan blue assays in SW480 cells transfected with NC or siG6PD, after 24 h incubation in complete medium or glucose-deprived medium. (C) Cell apoptosis assessed using flow cytometry by using Annexin V/propidium iodide (PI) staining. (D-E) Cell migration (D) and invasion ability (E) detected by Transwell assays in SW480 cells transfected with NC or siG6PD, after incubation in complete medium or glucose-deprived medium for 24 h. Representative images from triplicate experiments are shown. Magnification (×200). (F) Protein expressions of E-cadherin, vimentin and G6PD as determined by Western blotting in SW480 cells after transfection with NC or siG6PD, after incubation in complete medium or in glucose-deprived medium for 24 h. GAPDH was used as a loading control. Data are shown as mean ± SEM; n = 3. ∗*p* < 0 05, ∗∗*p* < 0 01, and ∗∗∗*p* < 0 001.

Through in vitro experiments, we also found that G6PD knockdown by siRNA significantly increased the sensitivity of SW480, SW620 and DLD-1 cells to glucose deprivation, leading to a significant increase in cell death and apoptosis (**Fig. 7A-C and S4**). Moreover, knockdown of G6PD in CRC cells not only inhibited migration and invasion but also reversed the increase in migration and invasion promoted by glucose deprivation (**Fig. 7D and 7E**). Subsequently, SW480 cells were transfected with siG6PD for 48 h and then treated with glucose-free medium or normal medium for 24 h. As illustrated in** Fig. 7F**, G6PD protein levels were higher in cells cultured under glucose deprivation than with normal medium, and its expression was decreased after transfection with siG6PD. Moreover, glucose deprivation-induced EMT (indicated by decreased E-cadherin and increased vimentin expression) was reversed by G6PD knockdown (**Fig.7F**). These results support that G6PD plays an important role in glucose deprivation-induced cell death, migration and invasion in CRC cells.

### Trx-1 and G6PD inhibitors suppress tumor growth of CRC xenografts* in vivo*

**Figure 8:**
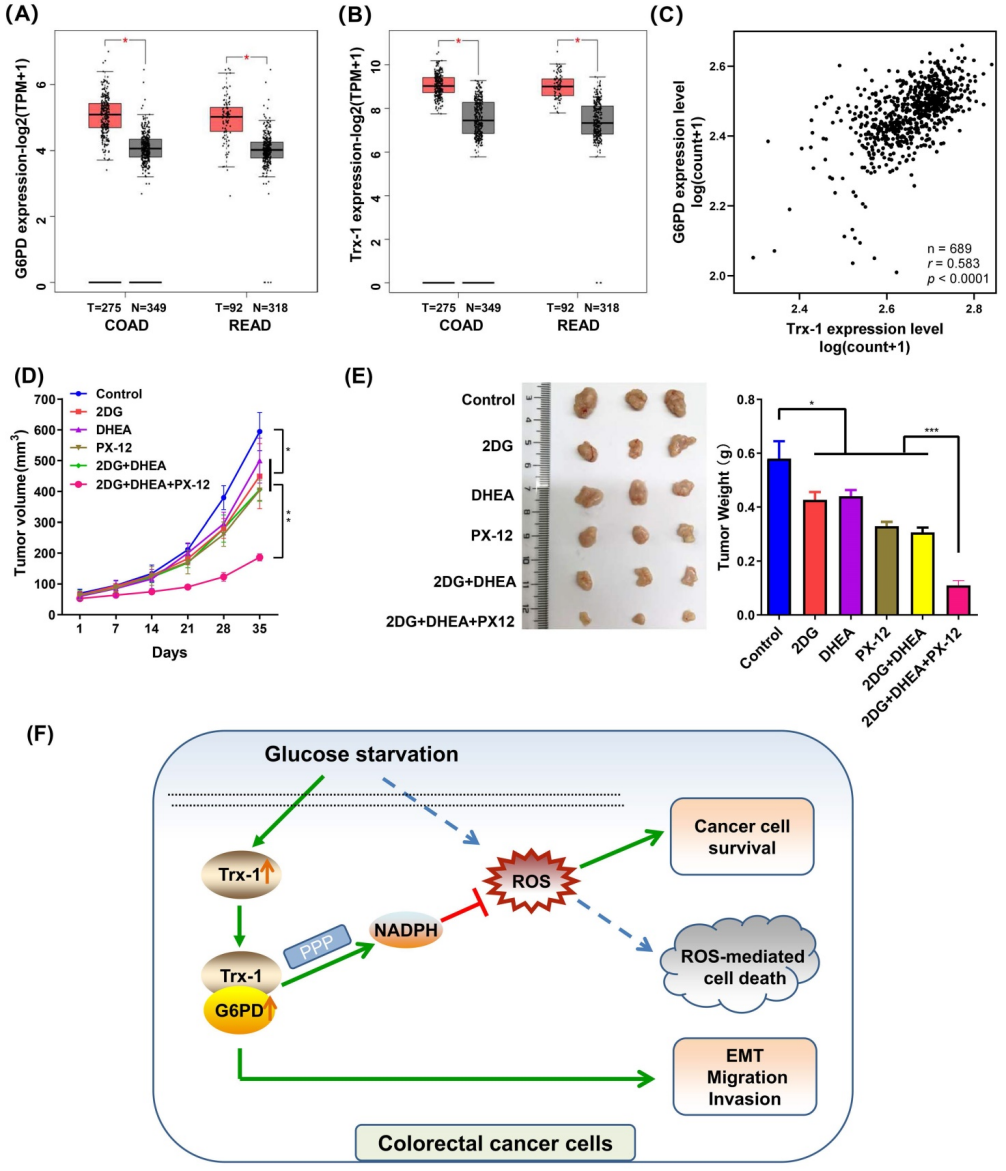
Inhibition of Trx-1 and G6PD suppresses colorectal carcinoma xenograft tumor growth* in vivo*. (A) Differential expression of G6PD in colorectal cancer tissues and normal tissues in both colon adenocarcinoma (COAD) and rectum adenocarcinoma (READ) from the GEPIA database. (B) Differential expression of Trx-1 in colorectal cancer tissues and normal tissues in both COAD and READ from the GEPIA database. (C) Correlation between Trx-1 and G6PD expression from analysis of The Cancer Genome Atlas (TCGA) dataset. (D) *In vivo* growth rate of colorectal carcinoma xenograft tumors. SW480 cells were injected subcutaneously into nude mice. After seven days, mice were randomly divided into six treatment groups (n = 5): Control, 2DG, DHEA, PX-12, 2DG + DHEA, and 2DG + DHEA + PX-12. Tumor volume was measured every three days (n = 5). (E) Three representative images of xenograft tumors (left panel) and final tumor weight measurement after treatment (right panel, n = 5). (F) A schematic diagram showing that Trx-1 supports colorectal cancer cell survival and promotes migration and invasion under glucose deprivation.

Firstly, we elucidated the expression of G6PD and Trx-1 in CRC and normal tissues using the public database GEPIA2 25. As depicted in **Fig. 8A and 8B,** both genes exhibited higher expression in CRC tumor tissue than in normal tissue. Correlation analysis of the expression profiles of these two genes among 689 CRC patients in TCGA database further revealed G6PD expression level to correlate positively with Trx-1 expression level (*p* < 0.0001, *r* = 0.596) (**Fig. 8C**). To explore the effects of Trx-1 and G6PD inhibition on tumor growth, we used dehydroepiandrosterone (DHEA, an inhibitor of G6PD) 29, 1-methylpropyl 2-imidazolyl disulfide (PX-12, an inhibitor of Trx-1) 30 and combination with 2DG to treat CRC xenografts in nude mice. 2DG, is an artificial analog of glucose, which can compete with glucose and deplete cellular energy 31. As shown in **Fig.8D and 8E**, treatment with any of the three compounds alone (2DG, DHEA, or PX-12) significantly suppressed tumor growth *in vivo*. Surprisingly, the combination regimen of 2DG, DHEA and PX-12 exhibited a stronger tumor inhibitory effect than any individual compound alone (**Fig. 8D and 8E**). Also, as shown in **figure S5**, the combination of 2DG, DHEA, and PX12 showed a stronger growth inhibitory effect than either agent alone *in vitro*. It is worth noting that Trx-1 interacts with G6PD and regulates the PPP in the event of glucose starvation and so constitutes a concise mechanism for the metabolic stress sensitivity of CRC cells (**Fig. 8F**).

## Discussion

Adaptation to the tumor microenvironment is critical for tumor cell survival. In this study, we demonstrate a novel mechanism by which CRC cells can survive in conditions of limited glucose availability. We report for the first time that Trx-1 regulates the activity of G6PD by interacting with it to regulate NADPH homeostasis and maintain tumor cell survival. We also found that both Trx-1 and G6PD are required for CRC cell migration, invasion and EMT under conditions of glucose starvation.

Our previous studies have reported that Trx-1 plays a key role in CRC growth and metastasis 10, 11. We investigated whether the survival of CRC cells under nutritional deprivation requires a large amount of Trx-1. We compared the cell death rate and clone formation ability under glucose deprivation depending onto the expression status of Trx-1. Indeed, overexpression of Trx-1 under glucose deprivation increased the survival rate of cancer cells, while Trx-1 knockdown significantly sensitized CRC cells to glucose deprivation-induced cell death and inhibited glucose deprivation-induced migration and invasion of CRC cells. Recently, conditional synthetic lethality has gained attention as an anticancer strategy 32. Our results suggest that knocking down Trx-1 may inhibit both cell survival and metastasis upon glucose deprivation, making it a potential target for metabolic intervention in clinical therapy.

The metabolism of tumor cells inevitably produces high levels of reactive oxygen species (ROS) due to oncogenic signaling and the high proliferation rate of tumor cells 33. Since oxidative stress (but not ATP deficiency) is the main trigger of cell death in metabolic stress, excessive ROS are toxic to tumor cells. Not surprisingly, they have evolved ways to deal with excess ROS, including activation of redox sensitive transcription factors, up-regulation of antioxidant enzymes, and induction of mitosis 34. Accordingly, we measured ROS changes in cultures with glucose deprivation compared to normal cultures, and found that glucose deprivation significantly increased the levels of ROS. Overexpression of Trx-1 effectively decreased the levels of ROS, while its knockdown dramatically increased the levels of ROS, which helped us to further investigate the metabolic relationship between the abundance of Trx-1 and cell survival under metabolic stress.

To investigate potential mechanisms of action of Trx-1 under glucose deprivation, we identified proteins that interact with Trx-1 using the HuPort^TM^ human protein chip. We found that the interactors of Trx-1 were significantly related to the “Metabolic Pathway”, and the protein with the highest enrichment was G6PD, a key control point of the PPP. Further co-IP and immunofluorescence co-localization analysis all provided evidence for an interaction between these two proteins. Under conditions of glucose deprivation, knockdown of Trx-1 effectively inhibited G6PD expression and activity, whereas its overexpression increased G6PD expression and activity. The complex regulation of G6PD has been reported that glycosylation of G6PD increases G6PD activity 35, 36. In addition, high glucose was reported to induce the ubiquitylation of G6PD by VHL, an E3 ubiquitin ligase, thereby promoting the degradation of G6PD in podocytes 37. In our study, we found that Trx-1 and G6PD could bind to each other and the binding was enhanced when glucose was removed from the culture medium. Moreover, overexpression or knockdown of Trx-1 had no effect on G6PD mRNA expression in SW480 cells (data not shown). Therefore, we speculate that the interaction of Trx-1 and G6PD may suppress G6PD ubiquitylation and degradation, and increase G6PD protein expression. These changes in G6PD activity are the result of changes in G6PD protein expression. However, further studies are needed to confirm this hypothesis.

Glucose in tumor cells mainly provides biomass by aerobic glycolysis and by entrying PPP, an important metabolic pathway for NADPH production. Maintaining an adequate amount of NADPH is essential for cancer cells to survive under oxidative stress 17. G6PD catalyzes the first step of PPP and is a key enzyme that generates NADPH to maintain reduced GSH, which scavenges ROS and protects cancer cells from oxidative damage 17, 21. Indeed, many oncogenic signaling networks are involved in the regulation of PPP flux and NADPH homeostasis. For example, TIGAR, a p53-inducible gene, decreases glycolytic activity and promotes PPP 38. In contrast, p53 itself inhibits the PPP rate-limiting enzyme G6PD, and loss of p53 function increases glucose flux to PPP 39. Activation of LKB-AMPK also prolongs tumor survival under metabolic stress by inhibiting NADPH consumption 40. We overexpressed and knocked down Trx-1 in cells to investigate the relationship between Trx-1 and NADPH homeostasis. Our results show that Trx-1 plays an important role in maintaining NADPH production.

To survive in an unfavorable microenvironment, cancer cells must adapt and move to sites with better growth conditions 41. Therefore, exposure to a restricted microenvironment leads to clonal selection of cancer cells and the development of a more malignant phenotype 42. For tumor invasion and metastasis to occur, cancer cells must adapt to a stressed microenvironment characterized by oxygen or nutrient deprivation, local acidosis, and increased ROS 43. Aggressive growth and metastatic spread are hallmarks of malignant tumors and result in high mortality rates in cancer patients 44. Moreover, EMT, a highly plastic transformation of epithelial cells into mesenchymal phenotypes, has been reported to be associated with aggressive or metastatic phenotypes during cancer progression 45. Our results showed that glucose deprivation induced the migration, invasion, and EMT of CRC cells and that knockdown of Trx-1 or G6PD could reverse the migration, invasion, and the EMT process induced by glucose deprivation. These results suggest that both Trx-1 and G6PD play important roles in glucose deprivation induced migration, invasion, and EMT in CRC cells. Moreover, we found that the combined inhibition of Trx-1, G6PD, and glycolysis produced a substantial anti-tumor effect in CRC xenografts *in vivo*.

In this study, we confirmed that the Trx-1 expression is critical for survival and metastasis of CRC cells in a metabolic stress microenvironment. Inhibition of Trx-1 expression by shRNA increases glucose starvation-induced cell death and inhibits migration and invasion. Furthermore, proteome chip and co-IP experiments identified and confirmed an interaction between Trx-1 and G6PD. Knockdown of Trx-1 can decrease G6PD protein expression and activity, thereby reducing NADPH production, increasing ROS levels, and enhancing glucose starvation-induced cell death, suggesting that Trx-1 may regulate PPP via G6PD. Thus, our results have revealed another mechanism for regulating energy metabolism in CRC cells and provided potential metabolic intervention targets for clinical treatment.

## Supplementary Material

Supplementary figures.
