# Thioredoxin 1 supports colorectal cancer cell survival and promotes migration and invasion under glucose deprivation through interaction with G6PD: Erratum

**DOI:** 10.7150/ijbs.94220

**Published:** 2024-02-01

**Authors:** Fengying Lu, Daoquan Fang, Shuhan Li, Zuyue Zhong, Xiujiao Jiang, Qinqin Qi, Yining Liu, Wenqi Zhang, Xiaohui Xu, Yangyang Liu, Weijian Zhu, Lei Jiang

**Affiliations:** 1 Central Laboratory, the First Affiliated Hospital of Wenzhou Medical University, Wenzhou 325000, China.; 2 Changzhou maternal and Child Health Care Hospital, Changzhou Medical Center, Nanjing Medical University, Changzhou, 213000, China.

In our paper, the authors identified some errors in Figures. Specifically, the incorrect representative images for the transwell invasion results (glucose- group of SW480 cells) in Fig.1D and the transwell migration results (glucose- group of SW480-shLuc cells) in Fig.5B were used when typesetting the pictures. In addition, the incorrect control (glucose+/NAC- group) was used for analyzing apoptosis of SW620 cells in Fig.2C, and the SW480 cells with glucose+/NAC- group and glucose+/NAC+ group should be swapped because they were placed in the wrong place. The corrected figures are as follows. Data processing and statistical analysis were also updated accordingly.

Some descriptions of methods also need to be corrected: On page 5541, paragraph 2, lines 4-8 is corrected to “Cell apoptosis was determined using an Annexin V-FITC/propidium iodide (PI) apoptosis kit or an Annexin V-APC/7-AAD apoptosis kit (Multisciences Biotech Co., Ltd., China) according to the manufacturer's instructions.”

The authors apologize for these errors and state that the correction does not have any effect on the final conclusions of the paper.

## Figures and Tables

**Figure 1:**
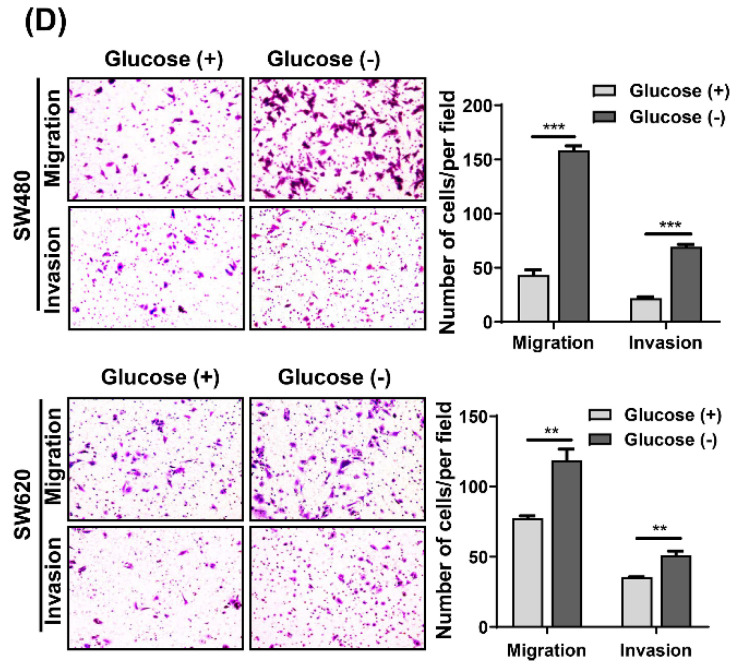
Corrected figure for original Figure 1D.

**Figure 2:**
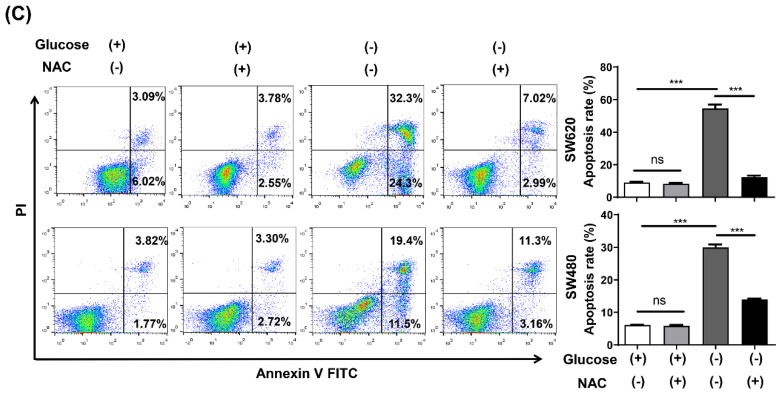
Corrected figure for original Figure 2C.

**Figure 5:**
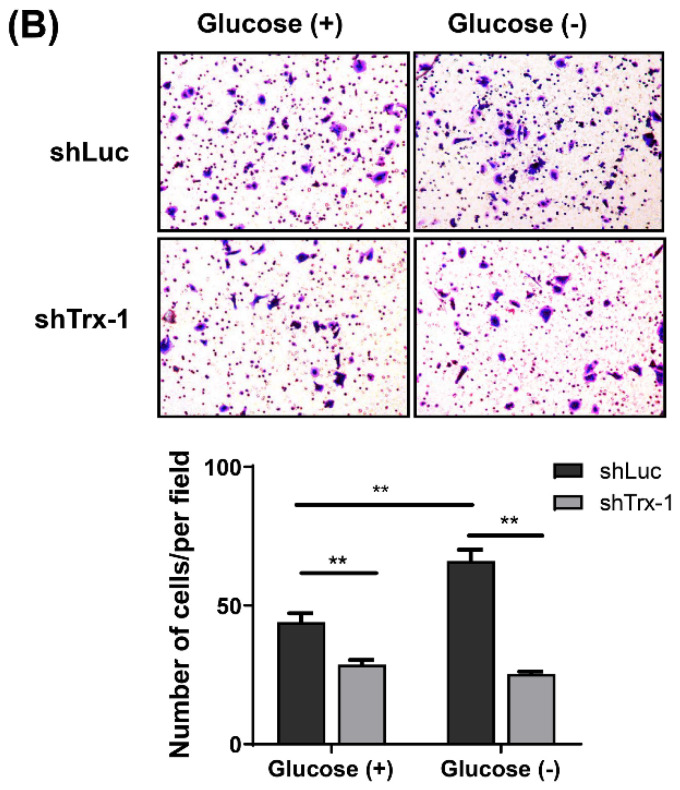
Corrected figure for original Figure 5B.

