# Supplementary material for: Thioredoxin 1 supports colorectal cancer cell survival and promotes migration and invasion under glucose deprivation through interaction with G6PD
Source: Int J Biol Sci. 2022 Aug 29;18(14):5539–53. doi: 10.7150/ijbs.71809 (PMC9461668; doi:10.7150/ijbs.71809)
Supplement: Supplementary file 1 — Supplementary figures. [file ijbsv18p5539s1.pdf]

Supplemental data

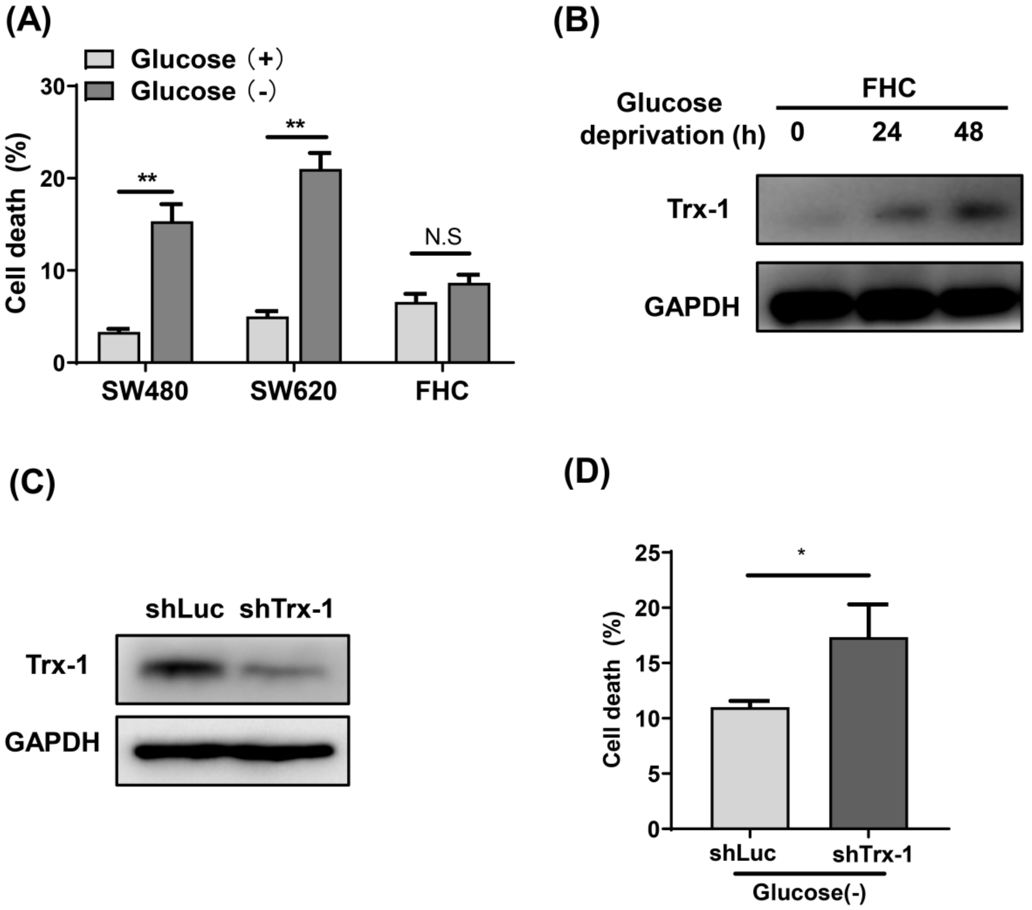

Figure S1 Toxicity of glucose deprivation in normal (FHC) cells. (A) Cell death assessed by trypan blue assays in SW480, SW620 and FHC cells grown for 24 h without glucose. (B) Trx-1 protein expression detected by Western blotting in FHC cells incubated for 24 h and 48 h in glucose-free medium. (C) Trx-1 protein expression detected by Western blotting in FHC cells transfected with shLuc or shTrx-1. (D) Cell death detected by the trypan blue assays in FHC cells transfected with shLuc or shTrx-1 for 24 h incubated in glucose-free medium. $*p <$ 0 05.

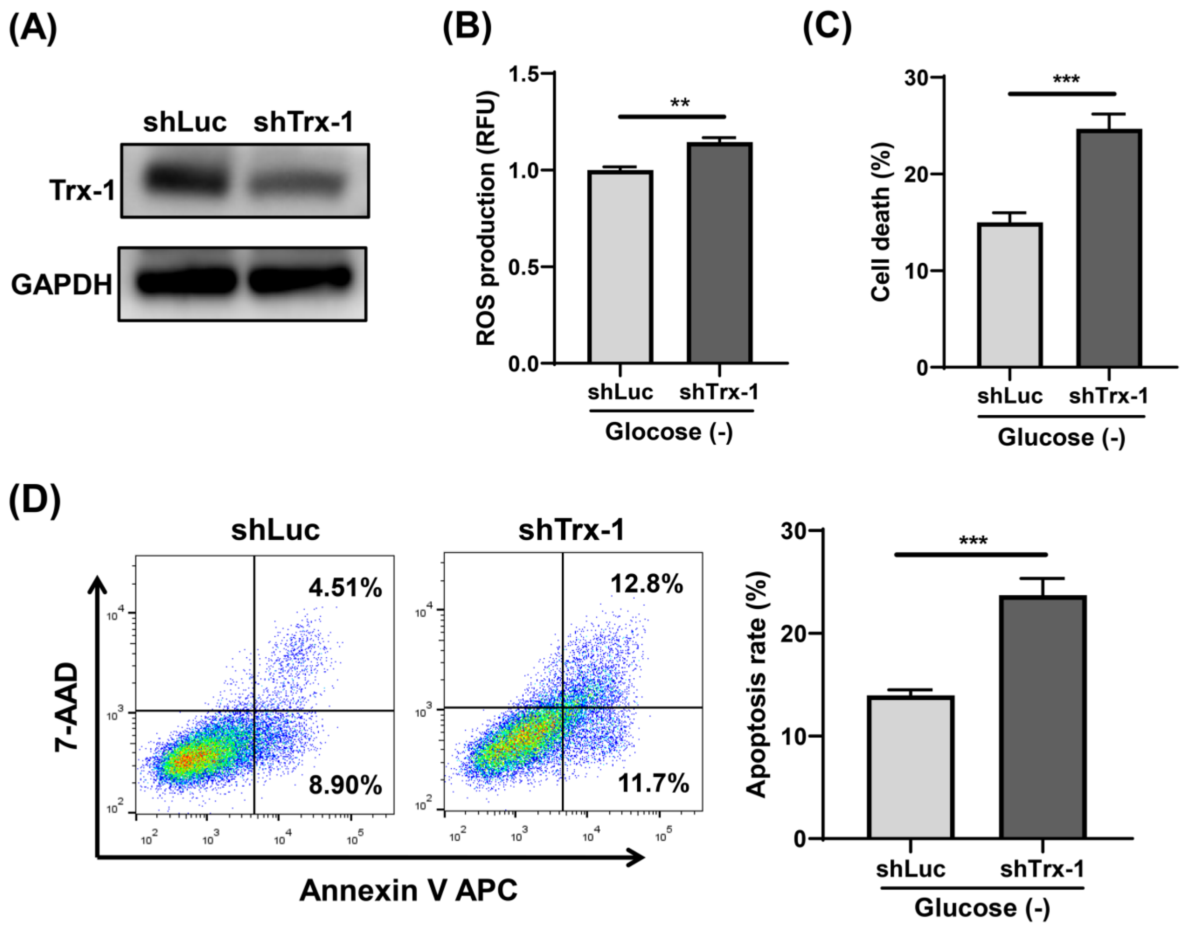

Figure S2 Knockdown of Trx-1 sensitizes colorectal cancer cells to glucose deprivation-induced cell death. (A) Trx-1 protein expression detected by Western blotting in SW620 cells stably expressing shLuc or shTrx-1. (B) The ROS level in SW620 cells stably expressing shLuc or shTrx-1 incubated in glucose-deprived medium for 24 h. (C) Cell death in SW620 cells stably expressing shLuc or shTrx-1 incubated for 24 h in glucose-free medium was determined using the trypan blue assays . (D) Cell apoptosis assessed by flow cytometry with Annexin V staining in SW620 cells stably expressing shLuc or shTrx-1 incubated in glucose-deprived medium for 24 h. $**p < 0\ 01$, and $***p < 0\ 001$.

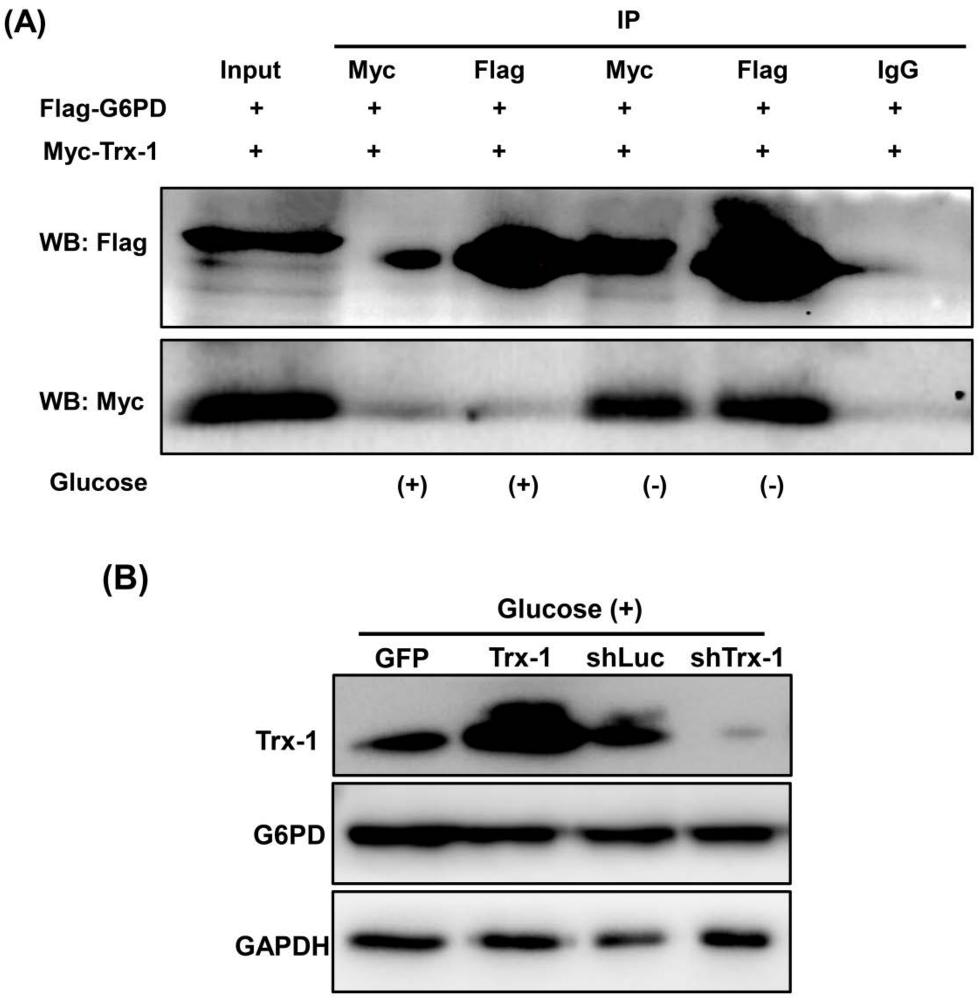

Figure S3 The interaction between Trx-1 and G6PD in colorectal cancer cells. (A) Co-immunoprecipitation (Co-IP) analysis of the interaction between Trx-1 and G6PD in SW480 cells transfected with Flag-G6PD and Myc-Trx-1 in the absence and presence of glucose. (B) Protein expressions of G6PD and Trx-1 determined by Western blotting in SW480 cells stably expressing GFP, Trx-1, shLuc or shTrx-1 in normal medium.

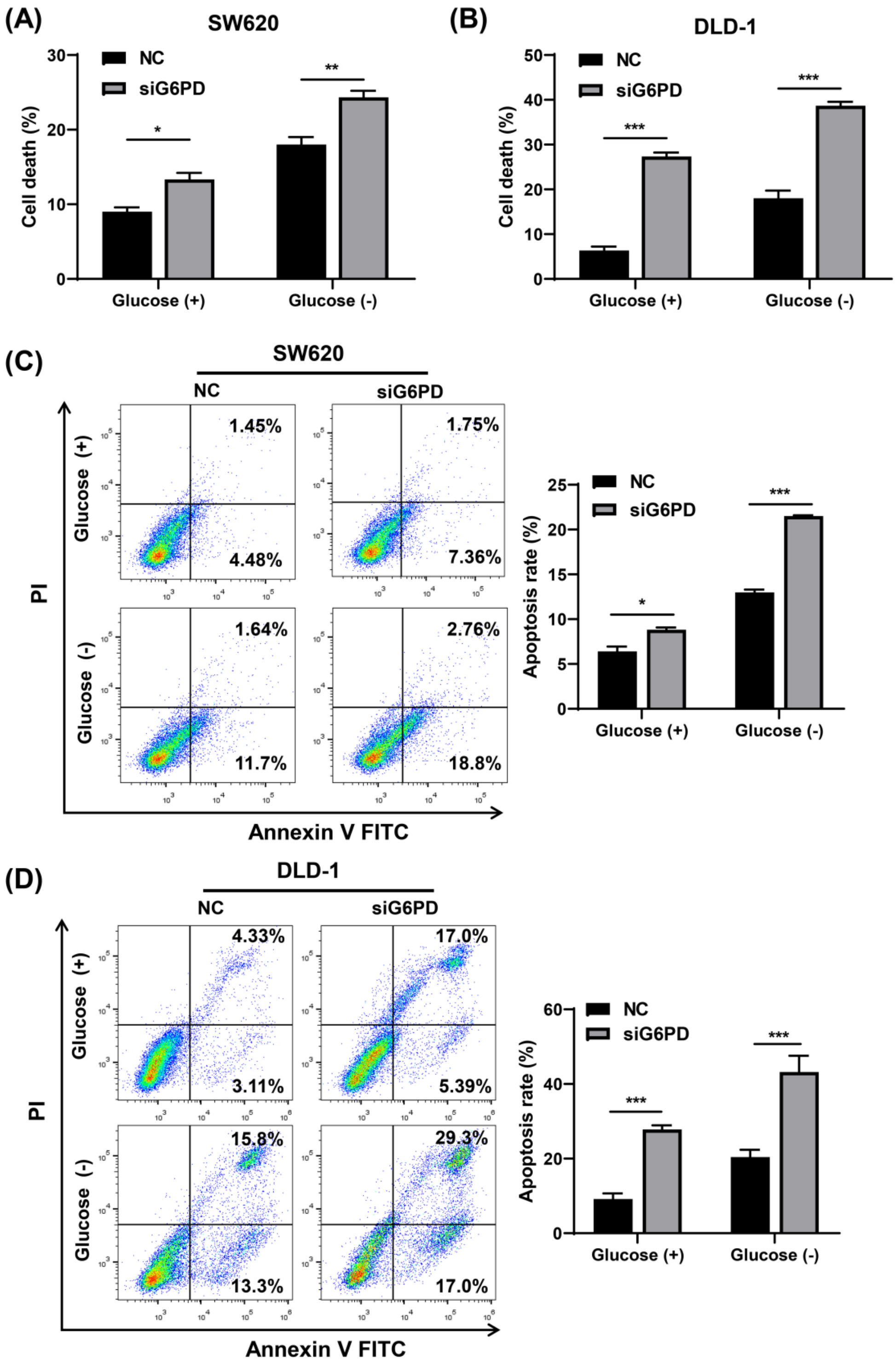

Figure S4 Knockdown of G6PD sensitizes colorectal cancer cells to glucose deprivation-induced cell death. (A-B) Cell death assessed by trypan blue assays in SW620 and DLD-1 cells transfected with NC or siG6PD, after 24 h incubation in complete medium or glucose-deprived medium. (C-D) Cell apoptosis assessed by flow cytometry using

Annexin V/propidium iodide (PI) staining in in SW620 and DLD-1 cells transfected with NC or siG6PD, after 24 h incubation in complete medium or glucose-deprived medium.

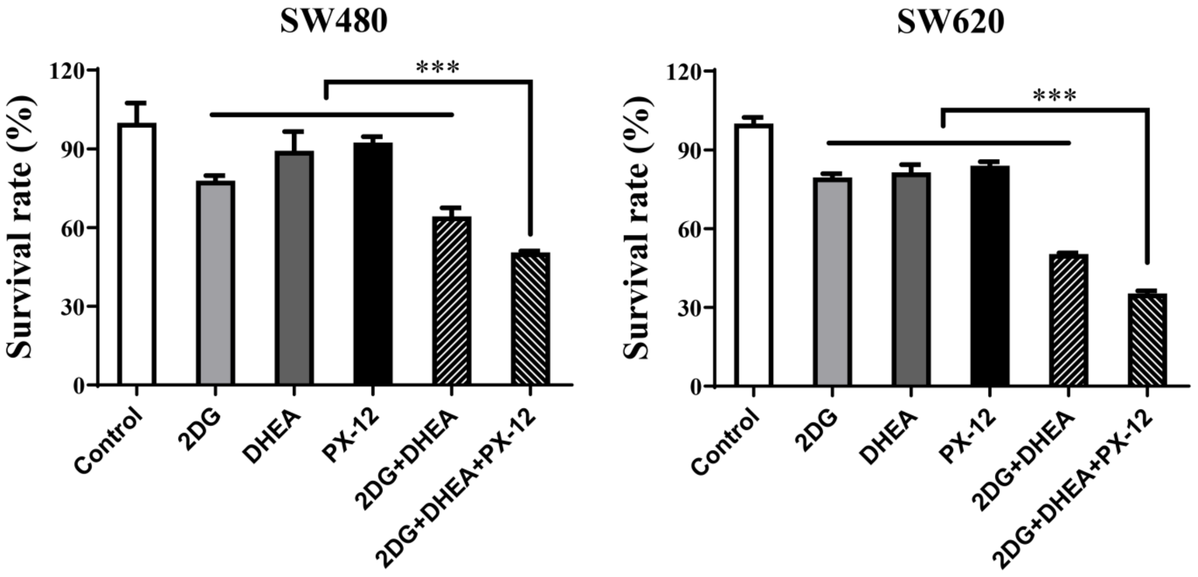

Figure S5 Combined treatment with PX12, DHEA, and 2DG increases cytotoxicity in human colorectal cancer cells. SW480 and SW620 cells were treated with PX12 (2.5 μM), DHEA (75 μM), 2DG (2.5 mM), or the combination for 24 h, and cell viability was measured by CCK-8 assay.
